# Temporal dynamics of ferroptosis markers and neuroprotective intervention in cerebral ischemia-reperfusion injury: Insights for therapeutic strategy development

**DOI:** 10.22038/ijbms.2025.82625.17865

**Published:** 2025

**Authors:** Shuangyan Bao, Chaojie Liu, Qian Wang Wang, Chenggui Zhang, Hairong Zhao

**Affiliations:** 1 Yunnan Provincial Key Laboratory of Entomological Biopharmaceutical R&D, College of Pharmacy, Dali University, Dali, Yunnan, PR China; 2 National-Local Joint Engineering Research Center of Entomoceutics, Dali, PR China

**Keywords:** Blood-brain barrier Ferroptosis, Ischemic stroke, Lipid peroxidation Oxidative stress

## Abstract

**Objective(s):**

This study examines the temporal dynamics of ferroptosis following cerebral ischemia-reperfusion injury (CIRI) to establish a theoretical framework for innovative therapies that enhance neuronal survival by mitigating ferroptosis.

**Materials and Methods:**

An experimental CIRI model was established in mice via middle cerebral artery occlusion and reperfusion (MCAO/R). Behavioral assessments were conducted, and blood-brain barrier (BBB) integrity was evaluated using transmission electron microscopy. Immunoblotting and ELISA were performed to determine ferroptosis dynamics post-MCAO/R. Additionally, CIRI mice received intraperitoneal injections of Ferrostain-1 (10 mg/kg/d) and Erastin (30 mg/kg/d). The effects of ferroptosis on CIRI were further verified through 2,3,5-Triphenyltetrazolium chloride and hematoxylin-eosin staining.

**Results:**

MCAO/R induced BBB disruption, and was associated with a reduction in GSH activity (at 1, 3, and 5 days), elevated Fe(2+) levels (at 1 day), as well as decreased MDA levels (at 3 days). Concurrently, ferroptosis markers, including NRF2, xCT, and GPX4, were significantly down-regulated on day 1, reaching their nadir by day 3, whereas HO-1 exhibited an inverse trend. Notably, Ferrostatin-1 pretreatment conferred a protective effect against CIRI, in contrast to the MCAO and Erastin groups.

**Conclusion:**

This study elucidates the temporal dynamics of ferroptosis markers in the early stages of stroke, highlighting a therapeutic window for ferroptosis-related CIRI. These findings underscore the importance of targeting ferroptosis to improve neuronal survival and inform future CIRI therapies.

## Introduction

Stroke is a significant public health concern worldwide, as the third leading cause of death and the first leading cause of disability, which seriously affects human physical and mental health and quality of life (1, 2). A lack of blood flow causes ischemic stroke (IS); the incidence of IS is higher than that of hemorrhagic stroke, accounting for approximately 87% (3, 4). The pathogenesis of IS is very complex and is not fully understood (5, 6). CIRI and blood flow interruption after ischemia lead to hypoxia and glucose deficiency, energy metabolism disorders, oxidative stress, inflammatory responses, glutamate excitotoxicity, and other complications. Apoptosis, autophagy, necrosis, and pyroptosis can ultimately lead to neuronal death (7, 8). Various signaling pathways were activated in these pathological transitions, and their targeted regulation could serve as a potential therapeutic strategy. The pathophysiological process of IS is complex, and it is crucial to elucidate its accompanying damage and signaling mechanisms for the development of targeted interventions. 

Dixon *et al*. were the first to describe ferroptosis in 2012 (9, 10). It is an iron-dependent cell death mechanism caused by the accumulation of lipid peroxides and related metabolites in the plasma membrane, as well as the depletion of polyunsaturated fatty acids (11, 12). Iron overload and a massive accumulation of lipid peroxides in cells define ferroptosis (12, 13). Cells accumulate iron after responding to stimuli. Excess iron ions eventually cause membrane lipid peroxidation via the Fenton reaction, resulting in mitochondrial damage, decreased function, and eventually, cell death (14, 15).

Ferroptosis is one of the mechanisms of ischemia-reperfusion injury, which is generally accompanied by ROS overproduction and iron accumulation after reperfusion. Excessive Fe (2+)-induced Fenton reaction (Fenton) produces hydroxyl radicals, which cause cytotoxicity (16, 17). Cerebral microvascular endothelial cell (BMVEC) dysfunction, ROS accumulation causes significantly increased macrophages, blood-brain barrier (BBB) damage, hemorrhagic transformation, and cerebral edema after cerebral infarction, and early neurological deterioration (18-20). Cerebral ischemia and reperfusion will cause brain damage, damage the integrity of the BBB, and sharply increase the iron content in the brain parenchyma, causing iron overload, lipid peroxidation, and other disorders of iron metabolism, which will eventually lead to cell iron death (21, 22). I/R leads to increased presynaptic glutamate release, decreased reuptake, increased free radical production, and promotes iron death. In 1990, Patt *et al*. conducted a study that indicated the involvement of iron in CIRI (23). Interestingly, brain iron has been linked to the severity of cerebral infarction, suggesting its role in ischemia-reperfusion injury (24). Furthermore, some studies have reported an association between iron overload-related death and CIRI; however, the dynamic monitoring of iron overload-related pathways after CIRI has not been investigated (19, 25). Therefore, we dynamically monitored the ferroptosis-related pathways after CIRI and clearly observed a dynamic process of ferroptosis, underscoring a therapeutic time window for ferroptosis-related CIRI. These insights provide a valuable theoretical foundation for developing novel CIRI treatment modalities.

## Materials and Methods

### Animals

Adult male C57BL/6 mice weighing 25-30 g (8-10 weeks) were supplied by the Dali University Laboratory Animal Center. They were habituated to a 12 hr light/dark cycle in a temperature-controlled environment (22-25 ^°^C) with a humidity of 40-70% and free access to food and water. All experimental procedures and animal housing in this study were approved by the Institutional Animal Care and Use Committee of Dali University, China (Animal ethics No.: DLU2017-0117).

### Middle cerebral artery occlusion-reperfusion (MCAO/R) model

The MCAO procedure was performed according to the method outlined by Zea Longa, with improvements previously reported (26-28). The mice were anesthetized with an intraperitoneal injection of 1.25% tribromoethanol. All surgical materials and instruments were sterilized before use. The internal common carotid artery, external carotid artery (ECA), and internal carotid artery (ICA)(29, 30) were carefully separated, and a small incision was made in the CCA. Blood flow was obstructed using a sterile nylon suture (No.: MSMC21B120PK50, Ruiwode Bio-technology Co., Ltd., China) within the middle cerebral artery from the ECA to the ICA. After 60 min, the suture was removed to restore blood flow, thereby inducing MCAO/R. The sham group underwent the same procedures except that the suture was not inserted. During the surgery, the body temperature of the mouse was maintained at 36.5±0.5 ^°^C. The neurological score was assessed immediately after surgery, when the animal had fully recovered from anesthesia. 

### Behavior tests

The longa test, rotarod test, and pole test helped assess the neurological function of mice subjected to MCAO, according to our previously developed protocols. Rotarod test: The mice were placed on a rotating rod for the rotarod test (Shanghai Xinruan Information Technology Co., Ltd., China), with speeds incrementally increasing from 4 rpm to 40 rpm. The duration for each mouse to maintain its grip on the rotating rod was recorded (maximum time: 120 sec). Pole test: the mice were placed on the tip of an 8 mm (diameter) ×50 cm (length) wooden pole with its head upward. The mouse then attempts to descend to the floor by turning its head downward; meanwhile, the total time it takes to reach the floor is recorded.

### TTC staining

The mice were equally divided into four groups: Sham, MCAO/R, MCAO/R+Erastain (30 mg/kg/day), and MCAO/R+Ferrostain-1 (10 mg/kg/day). Each group received daily treatment for three consecutive days. TTC staining was used to evaluate infarct size. The TTC solution was prepared in phosphate-buffered saline (PBS, pH 7.4) in the dark at 37 ^°^C immediately before use. For this assessment, the brain was rapidly isolated and sliced into 2-mm-thick coronal sections. Subsequently, the brain slices were stained with a 0.2% TTC solution at 37 ^°^C for 15 min and then fixed with 4% paraformaldehyde (PFA) for 24 hr. The infarct volume was analyzed using Image-Pro Plus version 6.0 image analysis software.

### Transmission electron microscopy (TEM)

Mice were transcardially perfused with PBS, followed by the brain being sectioned into 1 mm-thick slices and post-fixed in 2.5% glutaraldehyde in PBS. Under a dissection microscope, tissue punches were taken to capture the IL and CL peri-infarct areas in the cortex. Tissues were washed three times in PBS, then post-fixed in 1% osmium tetroxide with 1% potassium ferricyanide for one hour. Following three additional PBS washes, the pellet was dehydrated through a graded series of 30-100% ethanol, 100% propylene oxide, and then infiltrated in a 1:1 mixture of propylene oxide: Polybed 812 epoxy resin for one hour. After several changes of 100% resin over 24 hrs, the pellet was embedded in a final change of resin, cured at 37 ^°^C overnight, followed by additional hardening at 65 ^°^C for two more days. Ultrathin (70 nm) sections were collected on 200 mesh copper grids, stained with 2% uranyl acetate in 50% methanol for ten minutes, followed by 1% lead citrate for seven minutes. Sections were imaged using a JEOL JEM 1011 transmission electron microscope (Peabody, MA) at 80 kV.

### Determination of GSH/GSSG levels

The mice were deeply anaesthetized after 48 hr of I/R. The brains were collected in 1.5 ml EP tubes and homogenized with 500 µl 5% SSA (A610610-0050, DOJINGDO, Japan) solution. The samples were centrifuged at 8000× g for ten minutes, and the supernatant was transferred into a new tube. The supernatant was then diluted with ddH2O, and the concentration was adjusted to 0.5% with SSA. The concentration of GSH/GSSG was evaluated using the GSSG/GSH Quantification Kit II (G263, DOJINGDO, Japan). The absorbance was measured at 405 nm or 415 nm by Multiscan Spectrum (Multiskan Sky, Thermo, USA)

### Brain MDA measurement

A brain sample of 10-30 mg was collected into 1.5 ml microtubes and homogenized with 300 µl of an antioxidant PBS solution over an ice bath. The mixture was then centrifuged at 10,000×g for five minutes. MDA content in brain tissues was analyzed using the MDA Assay Kit (M496, DOJINGDO, Japan). The samples were added 200 µl of Lysis Buffer and mixed thoroughly with a vortex oscillator, then left for five minutes at room temperature. The 300 µl working solution was added to each microtubule and mixed thoroughly with a vortex mixer. After heating in a constant temperature bath at 95 ^°^C for 15 min, the sample was cooled in an ice bath for five minutes and centrifuged at 10,000 ×g for ten minutes. 200 µl of supernatant was added to a transparent 96-well plate. The absorbance at 532 nm was measured using a Multiscan Spectrum. The MDA concentration in the sample was calculated by the standard MDA curve.

### Iron assay

Additionally, the concentration of Fe (2+) in the mouse brain was determined using an Iron Assay Kit (I291, Dojindo Laboratories, Japan). Brain tissue was homogenized in cold PBS, and the supernatant was collected. The supernatant was then ultrasonicated for five minutes and centrifuged at 16,000×g for ten minutes to remove insoluble matter. The samples were divided into three parts: Fe (2+) detection, total Fe detection, and blank control. The sample for total Fe detection was supplemented with a reducing solution, and the remaining samples were supplemented with equal volumes of assay buffer. All samples were incubated for ten minutes at 37 ^°^C, then transferred into a 96-well plate and continued to incubate for an additional 60 min at 37 ^°^C. The iron content was measured using a Multiskan Spectrum (Multiskan Sky, Thermo, USA) at 593 nm.

### Western blotting

The total protein was separated using RIPA lysis buffer with 1 mM PMSF (HY-L081, Med Chem Express, USA) and a protease inhibitor cocktail (HY-K0010, Med Chem Express, USA) for 15 min, followed by 15 min of centrifugation at 12000 rpm at 4 ^°^C. Protein concentration was measured using a BCA protein assay kit (P0011, Beyotime, China). Protein was separated with SDS-polyacrylamide gel electrophoresis, and then transferred from the gel onto a polyvinylidene difluoride (ISEQ00010, Millipore, USA) membrane. After being blocked with 5% nonfat dry milk in Tris-buffered saline plus Tween-20 (TBST) for 1.5 hr at room temperature, the PVDF membranes were incubated overnight at 4 ^°^C with primary antibodies, followed by 1 hour at room temperature with secondary antibodies. The membranes were washed with TBST buffer, and the blots were visualized by Azure C300. The information of antibodies is as follows: NRF2 (sc-7269, Santa Cruz Biotechnology), GPX4 (ab216327, abcom), XCT (ab38898, abcom, Goat anti-Rabbit 0295G-SA, Bioss, China, Goat anti-Mouse, 0296G-SA , Bioss, China) HO-1(10701-1-AP, Proteintech, USA).

### HE staining

After collecting the tissues from each animal, the tissues were rinsed with water for two hours. After dehydration using different concentrations of ethanol, the tissues were dehydrated with xylene until they became transparent, embedded for one hour, and then sliced. Subsequently, the slides were roasted, dewaxed, hydrated, immersed in distilled water, and stained with an aqueous hematoxylin solution for three minutes, followed by differentiation with an ethanol-hydrochloric acid solution for 15 sec. After being slightly washed with water and blue returning solution for 15 sec, the slides were rinsed with water and stained with eosin for three minutes. Images were obtained using an inverted microscope (Zeiss, Oberkochen, Germany).

### Statistical analysis

All data were presented as means and standard deviations. Means were compared using independent sample t-tests or one-way ANOVA. All experiments were performed in triplicate. A *P*-value of <0.05 indicated a significant difference.

## Results

### MCAO/R leads to disruption of the BBB

**Figure 1:**
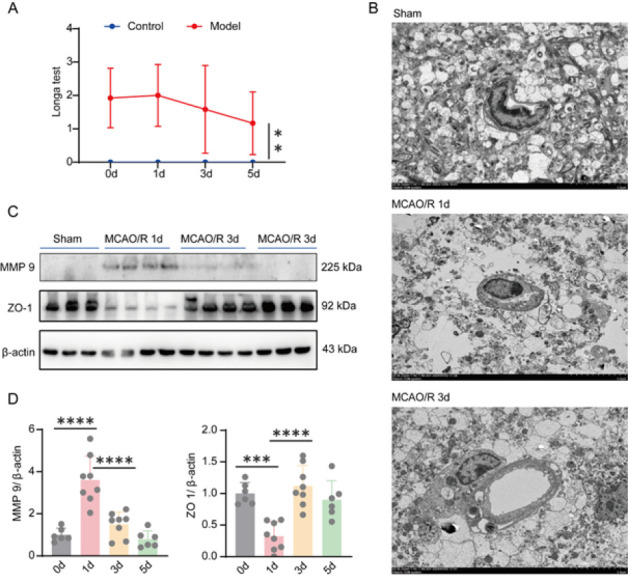
Middle cerebral artery occlusion and reperfusion leads to disruption of the blood brain barrier

The BBB plays a crucial role as a key component of the neurovascular unit, and its impairment during brain ischemic injury can significantly exacerbate cerebral damage. In this study, we employed transmission electron microscopy to investigate the structural alterations occurring at the BBB in mice subjected to MCAO/R. First, as shown in Figure 1A, the behavioral score of mice after thread implantation was significantly different from that of normal mice, which suggests the successful establishment of the CIRI model. Immediately, the results of the TEM showed that, the induction of MCAO/R led to the disruption of basal lamina, tight junction proteins, and endothelial cells (ECs), as well as mitochondrial swelling and pronounced expansion of astrocytic end-feet (Figure 1B). This result suggests that the BBB of the mice was damaged after MCAO.

In addition, compared to the Sham group, a substantial up-regulation of MMP9 expression was observed on the initial day post-MCAO/R, followed by a subsequent decrease in its expression levels. Conversely, the expression levels of ZO-1 demonstrated an initial decline on the first day post-MCAO/R, succeeded by a subsequent increase (Figures 1C and D). 

### MCAO/R leads to a decrease in GSH and Fe (2+) secretion and an up-regulation of MDA

**Figure 2:**
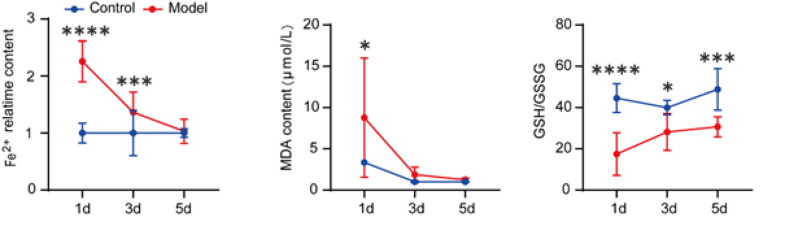
MCAO/R leads to a decrease in GSH and Fe (2+) secretion and an up-regulation of MDA

GSH/GSSH, MDA, and Fe (2+) serve as crucial biomarkers for assessing iron-induced cell death. This study scrutinized the variations in GSH/GSSH, MDA, and Fe (2+) levels in the brain tissues of mice at 1d, 3d, and 5d post-reperfusion (Figure 2). Compared to the Sham group, GSH/GSSG levels exhibited a down-regulation at the onset of reperfusion, followed by an up-regulation at three days. MDA, a pivotal end product of lipid peroxidation, has emerged as a key metric for assessing lipid peroxidation in iron-induced cell death. Notably, MDA levels in the brain tissues of mice spiked significantly at 1 day post-MCAO/R compared to the control group, subsequently demonstrating a marked recovery by the 3rd day and a return to normal levels by the 5th day. These findings underscore the peak oxidative stress incidence at the 24-hour mark post-MCAO/R. Furthermore, Fe (2+), a pivotal indicator of iron-induced cell death, exhibited substantial alterations on the first day, markedly deviating from the Sham group; by the fifth day, Fe (2+) levels no longer diverged from those of the Sham group, denoting a restoration to normal.

### Changes in MCAO/R-induced ferroptosis in mice

**Figure 3:**
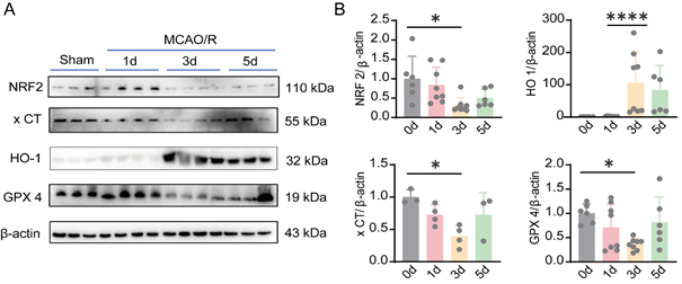
Changes in MCAO/R-induced ferroptosis in rats

Oxidative stress plays a crucial role in neuronal damage during CIRI. NRF2, GPX4, XCT, and HO-1 are the primary factors involved in oxidative stress and play key roles in ferroptosis. In our study, we assessed the changes in ferroptosis at the protein level on different days after MCAO/R. The results showed that, compared to the control group, the expression of NRF2, XCT, and GPX4 was significantly up-regulated on the first day and then decreased, whereas HO-1 exhibited the opposite trend (Figure 3).

### Inhibiting the onset of ferroptosis enhances neuroprotection in MCAO

**Figure 4:**
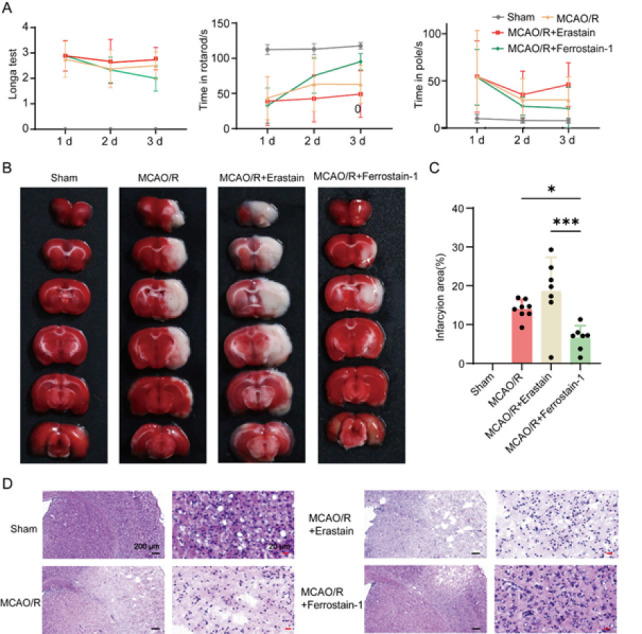
Inhibiting ferroptosis onset enhances neuroprotection in middle cerebral artery occlusion, as evidenced by reduced infarct volume and improved behavioral outcomes

As shown in Figure 4A, the behavioral score of the mice showed the successful establishment of the mouse MCAO model. Furthermore, compared with the MCAO/R+ erastin group and the model group, the behavior of mice in the MCAO/R+ ferrostain-1 group showed some improvement, indicating that cerebral ischemia and reperfusion damage in mice can be mitigated by inhibiting ferroptosis. Moreover, the results of TTC (Figure 4B and C) staining showed that, compared with the sham operation group, the infarct size was significantly larger in MCAO/R mice and was dramatically reduced in the MCAO/R+ ferrostain-1 group (*P*<0.05). Furthermore, mouse brain tissues were collected after sacrifice and stained with hematoxylin and eosin. No obvious pathological changes were observed in the sham group. As shown in Figure 4D, at 24 hr after MCAO, neuronal pyknosis and dissolution, cell body shrinkage, swelling of neurons and glial cells in the surrounding tissues of the infarction area, and enlargement of nerve cells and perivascular space were observed, which were milder in the MCAO/R+ ferrostain-1 group, but more aggravated in the MCAO/R+ erastin group. These results indicate that inhibiting the onset of ferroptosis enhances neuroprotection in MCAO.

## Discussion

Ischemic stroke has a high incidence, disability rate, mortality rate, recurrence rate, and economic burden, making it one of the major diseases threatening human health (31). Currently, the FDA has only approved intravenous tissue plasminogen activator and endovascular thrombectomy as treatment methods for IS. However, these two treatment methods must be used within 4.5 hr and 12 hr after a stroke, respectively, which is very urgent (32, 33). Reperfusion of blood after thrombus removal can also cause damage to patients, known as CIRI. However, the mechanism of injury caused by cerebral ischemia/reperfusion is complex and not fully understood, involving multiple signaling pathways (34-36). Therefore, clarifying the signaling pathways of cerebral ischemia-reperfusion injury is essential for improving CIRI.

Ferroptosis is one of the mechanisms that cause CIRI. Ferroptosis is a novel programmed cell death mode, characterized by excessive iron deposition inducing an increase in reactive oxygen species (ROS) within the cell, depletion of intracellular antioxidant glutathione (GSH), and a decrease in expression of glutathione peroxidase-4 (GPX4) that clears lipid ROS, leading to cellular toxicity (22, 37). The results of this experiment show a significant decrease in GSH and GPX4 after CIRI, indicating that ferroptosis injury occurred after CIRI. Furthermore, some studies have reported an association between over-iron death and CIRI. However, the dynamic monitoring of iron death-related pathways after CIRI has not been investigated (20, 19). Therefore, we dynamically monitored the ferroptosis-related pathways after CIRI and clearly observed a dynamic process of ferroptosis. This dynamic process further confirmed that the neuroprotection against cerebral ischemia and reperfusion injury can be strengthened by regulating the iron-related death pathway. This lays a solid theoretical foundation for the development of innovative therapeutic interventions for CIRI.

Finally, we further verify the link between ferroptosis and CIRI, TTC and HE results showed that ferroptosis inhibitors can improve the neurotoxicity of cerebral ischemia reperfusion, while ferroptosis inducer aggravated the neurotoxicity of cerebral ischemia reperfusion injury, further indicating that the neuroprotective effect of cerebral ischemia reperfusion injury can be realized by regulating the expression of ferroptosis pathway, which provides essential theoretical support to improve the survival rate of neuronal cells by inhibiting cell ferroptosis.

(A) The Longa test scores of the model group and the control group mice. (B) The TEM results of the sham surgery group and the model group of mouse brain tissues. (C) Results of MMP-9 and ZO-1 protein expression at different times post-MCAO/R. (D) The relative statistical graph of protein expression. Data are presented as mean±SD (n=6-9). ***P<0.001, ****P<0.0001 compared with sham group

MCAO/R: Middle cerebral artery occlusion and reperfusion; BBB: Blood-brain barrier; TEM: Transmission electron microscopy; MMP-9: Matrix metalloproteinase-9; ZO-1: Zonula Occludens Protein-1

The results of Fe (2+), MDA, and GSH/GSSG contents at different times after modeling in mice. Data are presented as mean±SD (n=6). *P<0.05, **P<0.01, ***P<0.001, ****P<0.0001 compared with the control group

MCAO/R: Middle cerebral artery occlusion and reperfusion; GSH: Glutathione; MDA: Malondialdehyde

(A) Different protein expression results at various time points after modeling. (B) Relative statistical chart of protein expression for Figure 3A. Data are expressed as mean±SD (n=5-9). P-values were calculated in comparison with the sham group using one-way ANOVA, with *P<0.05, ****P<0.0001

MCAO/R: Middle cerebral artery occlusion and reperfusion

(A) The Longa test scores, time in rotarod, and pole of the different groups. (B) The TTC staining results of the other groups. (C) Relative statistical chart of the area of cerebral infarction for Figure 4B. (D) Hematoxylin and eosin staining of mice brain tissues (×100 and ×400). Data are expressed as mean±SD (n= 7-8). P-values were calculated in comparison with the sham group using one-way ANOVA, with *P<0.05, ***P<0.001

MCAO/R: Middle cerebral artery occlusion

**Figure 5:**
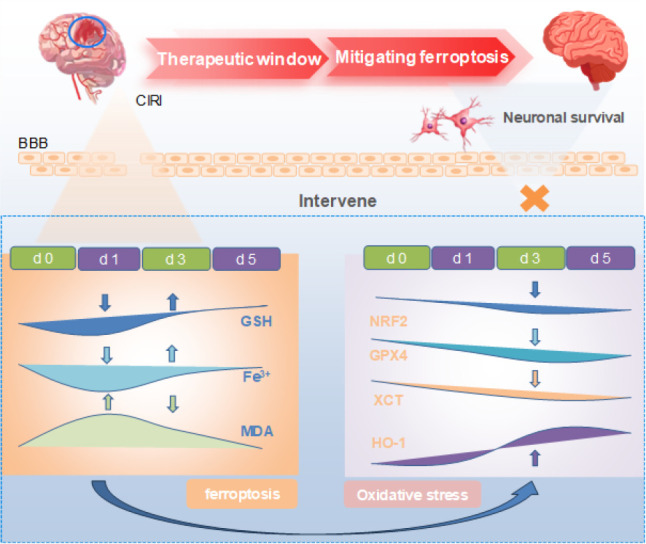
Graphical Abstract

Induction of MCAO/R resulted in the disruption of the blood-brain barrier (BBB). Concurrently, post-MCAO/R, mice displayed dynamic changes in the expression levels of GPX4, x-CT, NRF2, and HO-1. These changes initiated ferroptosis, which in turn exacerbated neuronal damage and cerebral infarction. By implementing targeted interventions within the optimal therapeutic window, ferroptosis can be suppressed, leading to improved neuronal survival

MCAO/R: Middle cerebral artery occlusion and reperfusion

## Conclusion

Overall, ferroptosis was closely related to CIRI (Figure 5). Dynamic monitoring of ferroptosis following brain ischemia-reperfusion injury revealed a specific therapeutic time window for ferroptosis-related CIRI, providing a theoretical basis for enhancing neuronal cell survival through ferroptosis inhibition.

## Data Availability

The research data in the current study are available from the corresponding author upon reasonable request.

## Declaration

We have not used any AI tools or technologies to prepare this manuscript.

## References

[1] Martins SO, Mont’Alverne F, Rebello LC, Abud DG, Silva GS, Lima FO (2020). Thrombectomy for stroke in the public health care system of Brazil. N Engl J Med.

[2] Thayabaranathan T, Kim J, Cadilhac DA, Thrift AG, Donnan GA, Howard G (2022). Global stroke statistics. Int J Stroke.

[3] Grysiewicz RA, Thomas K, Pandey DK (2008). Epidemiology of ischemic and hemorrhagic stroke: Incidence, prevalence, mortality, and risk factors. Neurol Clin.

[4] Barthels D, Das H (2020). Current advances in ischemic stroke research and therapies. Biochim Biophys Acta Mol Basis Dis.

[5] Sommer CJ (2017). Ischemic stroke: Experimental models and reality. Acta Neuropathol.

[6] Maida CD, Norrito RL, Daidone M, Tuttolomondo A, Pinto A (2020). Neuroinflammatory mechanisms in ischemic stroke: Focus on cardioembolic stroke, background, and therapeutic approaches. Int J Mol Sci.

[7] Liu R, Li Y, Zhang X, Tang K, Li J, Fang T (2024). Applications and mechanisms of taohong siwu decoction in the treatment of ischemic stroke. Pharmacogn Mag.

[8] Yang K, Zeng L, Yuan X, Wang S, Ge A, Xu H (2022). The mechanism of ferroptosis regulating oxidative stress in ischemic stroke and the regulation mechanism of natural pharmacological active components. Biomed Pharmacother.

[9] Dixon SJ, Lemberg KM, Lamprecht MR, Skouta R, Zaitsev EM, Gleason CE (2012). Ferroptosis: An iron-dependent form of nonapoptotic cell death. Cell.

[10] Yang L, Guttman L, Dawson VL, Dawson TM (2024). Parthanatos: Mechanisms, modulation, and therapeutic prospects in neurodegenerative disease and stroke. Biochem Pharmacol.

[11] Yuan H, Pratte J, Giardina C (2021). Ferroptosis and its potential as a therapeutic target. Biochem Pharmacol.

[12] Su LJ, Zhang JH, Gomez H, Murugan R, Hong X, Xu D (2019). Reactive oxygen species-induced lipid peroxidation in apoptosis, autophagy, and ferroptosis. Oxid Med Cell Longev.

[13] Dodson M, Castro-Portuguez R, Zhang DD (2019). NRF2 plays a critical role in mitigating lipid peroxidation and ferroptosis. Redox Biol.

[14] Yamada N, Karasawa T, Wakiya T, Sadatomo A, Ito H, Kamata R (2020). Iron overload as a risk factor for hepatic ischemia-reperfusion injury in liver transplantation: Potential role of ferroptosis. Am J Transplant.

[15] Shen Z, Liu T, Li Y, Lau J, Yang Z, Fan W (2018). Fenton-reaction-acceleratable magnetic nanoparticles for ferroptosis therapy of orthotopic brain tumors. ACS Nano.

[16] He YJ, Liu XY, Xing L, Wan X, Chang X, Jiang HL (2020). Fenton reaction-independent ferroptosis therapy via glutathione and iron redox couple sequentially triggered lipid peroxide generator. Biomaterials.

[17] Rochette L, Dogon G, Rigal E, Zeller M, Cottin Y, Vergely C (2022). Lipid peroxidation and iron metabolism: Two corner stones in the homeostasis control of ferroptosis. Int J Mol Sci.

[18] Li X, Ma N, Xu J, Zhang Y, Yang P, Su X (2021). Targeting ferroptosis: Pathological mechanism and treatment of ischemia‐reperfusion injury. Oxid Med Cell Longev.

[19] Wang P, Cui Y, Ren Q, Yan B, Zhao Y, Yu P (2021). Mitochondrial ferritin attenuates cerebral ischaemia/reperfusion injury by inhibiting ferroptosis. Cell Death Dis.

[20] Guo H, Zhu L, Tang P, Chen D, Li Y, Li J (2021). Carthamin yellow improves cerebral ischemiareperfusion injury by attenuating inflammation and ferroptosis in rats. Int J Mol Med.

[21] Lochhead JJ, Ronaldson PT, Davis TP (2024). The role of oxidative stress in blood–brain barrier disruption during ischemic stroke: Antioxidants in clinical trials. Biochem Pharmacol.

[22] Zhou Y, Liao J, Mei Z, Liu X, Ge J (2021). Insight into crosstalk between ferroptosis and necroptosis: Novel therapeutics in ischemic stroke. Oxid Med Cell Longev.

[23] Patt A, Horesh IR, Berger EM, Harken AH, Repine JE (1990). Iron depletion or chelation reduces ischemia/reperfusion-induced edema in gerbil brains. J Pediatr Surg.

[24] Zhang Q, Jia M, Wang Y, Wang Q, Wu J (2022). Cell death mechanisms in cerebral ischemia–reperfusion injury. Neurochem Res.

[25] Guan X, Li Z, Zhu S, Cheng M, Ju Y, Ren L (2021). Galangin attenuated cerebral ischemia-reperfusion injury by inhibition of ferroptosis through activating the SLC7A11/GPX4 axis in gerbils. Life Sci.

[26] Longa EZ, Weinstein PR, Carlson S, Cummins R (1989). Reversible middle cerebral artery occlusion without craniectomy in rats. Stroke.

[27] Kuge Y, Minematsu K, Yamaguchi T, Miyake Y (1995). Nylon monofilament for intraluminal middle cerebral artery occlusion in rats. Stroke.

[28] Zhao HR, Wang M, Gao Y, Wu XM, Xiao H, Yang DS (2022). Vespakinin-M, a natural peptide from Vespa magnifica, promotes functional recovery in stroke mice. Commun Biol.

[29] Zeng YQ, Hao L, Chen Y, Liu SY, Fan Y, Zhao ZH (2023). Optimizing intra-arterial hypothermia scheme for acute ischemic stroke in an MCAO/R rat model. Sci Rep.

[30] Wang Q, Liu CJ, Chen MR, Zhao J, Wang DX, Gao PF (2024). Mastoparan M promotes functional recovery in stroke mice by activating autophagy and inhibiting ferroptosis. Biomed Pharmacother.

[31] Khanevski AN, Bjerkreim AT, Novotny V, Næss H, Thomassen L, Logallo N (2019). Recurrent ischemic stroke: incidence, predictors, and impact on mortality. Acta Neurol Scand.

[32] Shi ZS, Loh Y, Walker G, Duckwiler GR (2010). Endovascular thrombectomy for acute ischemic stroke in failed intravenous tissue plasminogen activator versus non–intravenous tissue plasminogen activator patients: revascularization and outcomes stratified by the site of arterial occlusions. Stroke.

[33] Saver JL, Adeoye O (2021). Intravenous thrombolysis before endovascular thrombectomy for acute ischemic stroke. JAMA.

[34] Maida CD, Norrito RL, Daidone M, Tuttolomondo A, Pinto A (2020). Neuroinflammatory mechanisms in ischemic stroke: focus on cardioembolic stroke, background, and therapeutic approaches. Int J Mol Sci.

[35] Zhao Y, Zhang X, Chen X, Wei Y (2022). Neuronal injuries in cerebral infarction and ischemic stroke: From mechanisms to treatment. Int J Mol Med.

[36] Andrabi SS, Parvez S, Tabassum H (2020). Ischemic stroke and mitochondria: Mechanisms and targets. Protoplasma.

[37] Wang Z, Li Y, Ye Y, Zhu H, Zhang J, Wang H (2023). NLRP3 inflammasome deficiency attenuates cerebral ischemia-reperfusion injury by inhibiting ferroptosis. Brain Res Bullet.

